# Validation of Digital Applications for Evaluation of Visual Parameters: A Narrative Review

**DOI:** 10.3390/vision5040058

**Published:** 2021-11-24

**Authors:** Kevin J. Mena-Guevara, David P. Piñero, Dolores de Fez

**Affiliations:** 1 Group of Optics and Visual Perception, Department of Optics, Pharmacology and Anatomy, University of Alicante, San Vicente del Raspeig, 03690 Alicante, Spain; kjmg92@gmail.com (K.J.M.-G.); dolores.fez@ua.es (D.d.F.); 2 Department of Pathology, University Miguel Hernández, Sant Joan d’Alacant, 03550 Alicante, Spain; 3 Department of Ophthalmology, Vithas Medimar International Hospital, 03016 Alicante, Spain

**Keywords:** visual function, app, electronic device, visual acuity, tablet, contrast sensitivity

## Abstract

The current review aimed to collect and critically analyze the scientific peer-reviewed literature that is available about the use of digital applications for evaluation of visual parameters in electronic devices (tablets and smartphones), confirming if there are studies calibrating and validating each of these applications. Three bibliographic search engines (using the search equation described in the paper) and the Mendeley reference manager search engine were used to complete the analysis. Only articles written in English and that are evaluating the use of tests in healthy patients to measure or characterize any visual function aspects using tablets or smartphones were included. Articles using electronic visual tests to assess the results of surgical procedures or are conducted in pathological conditions were excluded. A total of 19 articles meeting these inclusion and exclusion criteria were finally analyzed. One critical point of all these studies is that there was no mention of the characterization (spatial and/or colorimetrical) of screens and the stimuli used in most of them. Only two studies described some level of calibration of the digital device before the beginning of the study. Most revised articles described non-controlled comparatives studies (73.7%), reporting some level of scientific evidence on the validation of tools, although more consistent studies are needed.

## 1. Introduction

The evaluation of visual function is crucial in the clinical practice of eye care professionals. This evaluation combines different tests characterizing the patient’s ability to perceive and integrate external light stimuli captured by sight, with proper coordination of both eyes’ visual systems [1,2]. In other words, the evaluation of visual function considers the optical eye system’s imaging process and brain processing of information. In the clinical setting, different aspects are evaluated in order to obtain complete information about the visual system status, such as visual acuity (VA), contrast sensitivity (CS), binocular vision (BV)—which in turn includes phorias, fusional vergences, or near point of convergence—accommodation, or color vision [3]. All these evaluations are performed by clinicians using different instruments and procedures that sometimes are time consuming and tiring for the patient.

In the current digital era, the use of computers, smartphones, tablets, and smartwatches is a typical daily practice, and there has been attempts to transfer theses usages to the eye care professional’s clinical practice [1,2]. Only in 2016 in Spain, the National Observatory of Telecommunications, and Information Society (ONTSI) reported that 85% of Spanish internet users with ages ranging from 16 to 65 used social media for an average of 1 h per day, and this included the usage of computers (91%), smartphones (95%), and tablets (48%) [4]. Likewise, digital applications (apps) for different purposes have increased exponentially in recent years [4]. Among these apps, those corresponding to the health field (e-health and m-health) are widely used [5]. Specifically, there are numerous apps for evaluating different aspects of visual function that can be easily accessed via the App Store (iOS) or Google Play store (android system). The use of these apps should be conducted with care as no information about the scientific validation of these tools is normally provided. Using non-validated apps in clinical settings to evaluate different aspects of the visual function may result in incorrect clinical decisions [6].

Furthermore, significant discrepancies in image reproduction are present among electronic devices. For example, previous research from our group has demonstrated large color reproduction differences between smartphones (Samsung Galaxy S4 and iPhone 4s), and tablets (Samsung Galaxy Tab 3 and iPad 4) [3]. Likewise, it has even been demonstrated that there are significant differences in digital reproduction of visual stimuli among different units of the same tablet model [7]. Therefore, it is worth asking to what extent is the use of applications available in digital stores correct with respect to evaluating visual function. Furthermore, in another recent study by our research group, the differences in luminance reproduction between 20 tablets (Samsung Galaxy Tab A, SM-T519, 2019 version), as well as their implications for contrast reproduction were evaluated, having as a result differences between the devices even if they are from the same manufacturing batch [8]. Although our study is based on applications for smartphones and tablets, it must be disregarded that validation tests are also being carried out on virtual reality devices, as reported by the study by Wroblewski et al. [9] that conducted a validation of the VirtualEye system (with its respective characterization of screens: luminance measurement).

It is necessary to define two concepts for a better understanding of the validation of the digital tools, the concepts of characterization and validation. Characterization is the procedure that allows us to determine peculiar attributes of an object in our study of a screen so that it is clearly distinguished from the others. Thus, the characterization ensures that the designed test is reproduced in each device, because each one can have a different reproduction. In contrast, validation is the procedure of providing firmness or certainty to an action or theory. In our study, clinical validation refers to confirming that the object of study (app) measures in a similar or comparable manner to the measures obtained with the gold standard (traditional test). Ideally, both concepts should be linked, but one does not exclude the other; they are complementary. If the screen of the device has not been previously characterized, how can you be ensuring that the comparison with the gold standard is reliable? At the very least, they cannot be considered to provide the same level of scientific evidence [8,10,11].

In addition, an important point to consider in this type of study is the privacy of the data, since these devices are using sensitive information of patients and must be properly treated, according to the European or American regulations’ General Data Protection Regulation (GDPR, 1995) and Protected Health Information (PHI) developed in the Health Insurance Portability and Accountability Act (HIPAA, 1996). This is clearly detailed in the scoping review conducted by Benjumea et al. [12] 2020 and Apple’s privacy section [13], where the regulations for the Health App are detailed as well.

The current review aimed to collect and analyze critically the scientific peer-reviewed literature that is available about the use of digital applications for the evaluation of visual parameters in electronic devices (tablets and smartphones), confirming if there are studies calibrating and validating each of these applications. To our knowledge, this is the first review on this crucial aspect: the validity of visual function evaluation using validated apps. It should be considered that some digital visual functions are being used in clinical investigations without confirming that they are adequate for such evaluations. Therefore, it is unknown if the validity of the results provided in these investigations are biased due to the use of non-validated digital clinical tools.

## 2. Materials and Methods

Three bibliographic search engines (the utilization of the search equation is described later in this paper) and the Mendeley reference manager search engine were used to complete the analysis. The following inclusion criteria were established in order to focus the search and to delimit the results:

- Articles showing the use of tests in healthy patients to evaluate any aspect of the visual function using tablets or smartphones;
- Articles written in English.

The exclusion criteria for the current review included articles using electronic visual tests to evaluate the results of surgical procedures or in pathological conditions, studies involving animals, and articles showing simulations or theoretical results. Before a more detailed analysis in pathological cases, we preferred to focus our analysis on healthy eyes, which is the most optimal situation as the potential bias of measurements may have a less relevant impact on clinical decisions. Future analysis of the literature should be performed in the future including pathological or post-surgical cases and considering the results of this previous analysis in healthy eyes, with the aim to compare possible differences.

The search equation used for this review was as follows.

*(“visual function” OR “visual acuity”) AND (“iPad” OR “app”) NOT (“Mice model” OR “Animal model”) NOT (“Amblyopia treatment” OR “ocular diseases”)*

The results obtained with this search equation was first filtered after reading the titles and abstracts and considering the inclusion and exclusion criteria defined. The articles’ complete text that passed this first filter was obtained and read to confirm their explicit inclusion or exclusion in the review. Finally, a qualitative assessment of the results obtained was performed after classifying them by subject.

## 3. Results

### 3.1. Search Results

**Figure 1:**
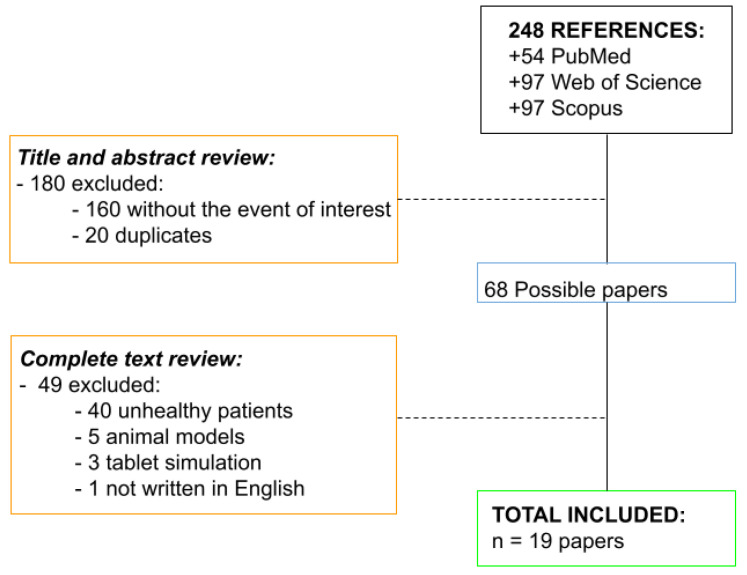
Flowchart showing the procedure followed in the current bibliographic review.

After searching on different platforms, a total of 248 articles were found (Figure 1). Specifically, 54 potentially useful results were found in the first search in PubMed (performed on 26 June 2019). On the same search date, 97 studies were found in the rest of the search platforms used.

Of all the articles found, 180 articles were excluded after verifying the first analysis described above for they did not meet the inclusion criteria. In the second analysis of the full text, 49 studies were excluded from the potentially eligible 68 articles (those previously selected).

### 3.2. Analysis of the Articles Included and Excluded

Table 1 summarizes the most relevant information of the articles finally included in the current article [2,6,14,15,16,17,18,19,20,21,22,23,24,25,26,27,28,29,30].

A total of 49 articles were excluded from the review. Most of them showed a comparison between digital measurements of parameters, such as visual acuity, contrast sensitivity, and reading speed, with traditional measurement methods, concluding that digital and traditional measures could not be used interchangeably.

The following causes of exclusion were found for the excluded articles: unhealthy patients and operated patients (40 works); analysis using animal models or human tissue (5 articles); articles not written in English; or use of tablet simulations (3 works). Specifically, of the 40 articles excluded due to unhealthy status or previous surgery, 25% were conducted in low vision patients, 17.5% in amblyopic patients, 12.5% in age-related macular degeneration (AMD) patients, and 7.5% in both diabetes and multiple sclerosis subjects. The rest of the excluded cases (2.5%) include other pathological conditions: maculopathy, stroke, retinoschisis, senile dementia, hemangiopericytoma, Parkinson, and albinism strabismus, blindness, or dry eye.

## 4. Discussion

Most of the articles finally included in this critical review of the existing scientific literature on the use of digital devices for evaluating visual function (57.9%) reported the use of evaluations of visual acuity: 10.5% for the assessment of reading speed, and 5.3% (each) for the evaluation of contrast sensitivity and color vision, respectively. Likewise, some articles report using digital applications to evaluate more than one aspect of the visual function, with 5.3% of studies studying the entire visual function, including healthy patients and those with ocular abnormalities. It is worth noting that, on average, the level of evidence reported in all of these investigations is limited, with 73.7% of them being comparatives studies, 5.3% being observational studies, and 5.3% being case reports. A cross-sectional study, a bibliographic review, and a clinical trial (blind examiner) were found and analyzed. More consistent studies should be designed and performed in order to validate the great variety of digital apps that are currently available for the evaluation of visual function. Comparative analyses in which the order of performance of tests (digital and traditional) is assigned randomly, with clear descriptions of the calibration process of screens and illumination conditions of the examination room, with an additional analysis of reliability, and with different examiners performing digital and traditional tests should be conducted. Indeed, at least one of these types of studies should have been conducted for any app that was released before indicating the possibility of its clinical use.

As mentioned, one critical point of all these studies is that there is no mention or partial description of the visualization conditions present during the measurements (observation distance, screen tilt, ambience illumination level, and screen brightness) or the characterization (spatial and/or colorimetrical) of screens and the stimuli used in most of them. Only the studies from de Fez et al. [2] and Rodriguez-Vallejo et al. [25] mentioned that a previous characterization of the digital device used had been performed. Likewise, it is not clear that digital apps evaluating contrast sensitivity have considered that the concept of contrast should be based on luminance and not on a concept of the difference of digital levels. The contrast defined considering the digital levels of a screen is not equivalent to the classical definition of contrast considering luminance, the definition used to calculate contrast sensitivity [3]. Therefore, in addition to improvement in the design of the clinical studies for evaluating the clinical usefulness of digital apps for assessing visual function, more information should be provided about the characterization of the screens in order to know if the differences obtained between digital and traditional tests may be due to problems of contrast and color reproduction of screens [3,7]. de Fez et al. [7] performed spatial and colorimetric characterization of different devices using different methods, demonstrating that mathematical adjustment methods such as gain-offset-gamma (GOG) adjustments provide worse results in color reproduction than methods based on 3D LUT tables. Furthermore, they find that another problem arises if a test designed colorimetrically for one device is presented on a different device. The digital levels of a stimulus calculated for a given device can produce different chromaticities when reproduced in another, because colorimetric characterization is device dependent. They found that on iPad devices, color reproduction errors are below the minimum level distinguishable by the human eye when using the device’s characterization data, but they can be more than six times higher if this is not performed. This fact implies that vision tests (color vision; CSF-Contrast Sensitivity Function) designed for a particular device can result in erroneous diagnoses when administered in other devices, even those of the same model. As an example, Black et al. [14] evaluated 85 patients in a blind examining clinical trial and found that the “Visual Acuity XL” application used on an iPad device provided significantly worse visual acuity measures (mean difference 0.18 logMAR) than those obtained with traditional methods. The authors did not mention that this difference may be attributed to screen reproduction errors or discrepancies in the stimuli’s conception and design.

Regarding the characterization of screens, it should be also mentioned that the reproduction of the chromaticity and/or the luminance of the stimulus may depend on its position due to the inhomogeneity of the screen (this is another part of spatial characterization). A full-screen stimulus may not be perceived as uniform due to this lack of homogeneity, and a stimulus at x degrees from the center may also be reproduced with different characteristics than the same stimulus at -x degrees. If only a small and constant area of the screen is to be used for the test in question, this part of the characterization can be omitted. In terms of color reproduction, colorimetry covers both chromaticity and luminance of color. A luminance-only characterization can be carried out if the stimulus is achromatic, but it should be considered that while a white stimulus has a single luminance value to be measured and characterized, an achromatic stimulus has gray levels. The use of a gamma curve is an approximation of luminance, which can be more or less reliable depending on the behavior of the particular device. For example, a gamma curve does not reproduce saturation at high power-on levels, as can happen on LCD and TFT screens. In contrast, the use of 3DLUT tables, which also approximates luminance values by interpolation can increase the reliability of the approximation [7,8].

As in the current review, Hogarty et al. [6] remarked in their bibliographic research the relevance of using properly validated digital applications for any type of praxis with patients, including the analysis of the entire visual function. This was a consistent conclusion from this previous review, as any of the digital applications revised in this literature search had proper validation. It should be noted that the use of non-validated digital apps for evaluating visual parameters in any research can result in inaccurate conclusions and subsequently to incorrect “scientific-based” clinical decisions. Indeed, new guidelines should be defined in the future in order to classify or identify, in Google play and Apple stores, whether an app can be used or not for clinical purposes according to scientific validations associated with this technology. A specific type of signaling or labelling in the store should be developed in order to avoid the inadequate use of apps in patients.

Despite the lack of information about the validation of apps and digital tests used in most of revised articles, some of them analyzed the clinical equivalences between digital and traditional tests, providing an evaluation or discussion of interchangeability between them. Azis et al. [27] found in a sample of 195 patients aged between 5 and 6 years that there was a good correlation between the results obtained using the “AAPOS Vision Screening” application and those obtained with conventional optotypes (ETDRS and Lea symbols). Specifically, the results obtained using the app (Lea symbols) to test visual acuity by parents as a potential screening tool were compared to gold standard vision testing by an optometrist using the Lea symbols chart. The authors concluded that the app evaluated could be considered as a promising tool for visual acuity screening among Malaysian preschool children. However, due to the specific type of population evaluated and the scarcity of information about the tablet used and its level of calibration, the results obtained cannot be extrapolated to the general population, but it can be considered as a first level of validation of this app. Concerning the measurement of visual acuity with digital devices, there is an additional aspect that must be considered. The minimum spatial detail available for some electronic devices given their pixel size does not allow measuring hyperacuity thresholds for close viewing distances. Therefore, many devices will not support visual acuity beyond 6/6 (1 min arc detail, e.g., VR goggles) and they will not allow measuring with reliability people with acuities down to 6/3 (0.5 min arc).

Rodríguez-Vallejo et al. [25] evaluated visual acuity and contrast sensitivity in 45 patients (Spanish adult university population) by employing a self-developed application that was previously validated [31]. These authors measured the chromaticity of an iPad with a retina display using the Spyder4Elite colorimeter and the luminosity of the room with theLX1330B luxmeter. Likewise, the brightness of the screen was set at the maximum. Concerning visual acuity, these authors compared the results of the visual acuity test of the app that was based on the ATS (Amblyopia Treatment Study) procedure and HOTV optotypes with the values obtained with the ETDRS chart projected on a screen (Optec 6500 system), obtaining a mean difference of approximately three letters on a logMAR chart with five letters per line. Moreover, the agreement between app and conventional test with respect to reliability was evaluated by citing 25 subjects a total of two or more sessions that are spaced a week apart. Coefficients of reliability were 0.15 logMAR for our method and 0.17 logMAR for the ETDRS testing protocol. In terms of contrast sensitivity, the mean differences between the app and the measures obtained with the Functional Acuity Contrast Test (FACT) were lower than 0.05 log units for all spatial frequencies. The limits of agreement between digital and traditional tests were higher for high spatial frequencies. According to these authors, this finding was explained by the lower repeatability of both tests for those spatial frequencies [25]. This study is one of the most complete validation of an app with respect to evaluating some visual function parameters and should be considered when trying to design a validation study for a specific app. They described the fact that the brightness of the screen was set on the maximum level (342 cd/m^2^) was a limitation of their study, which is over the recommended background luminance [25]. They describe as a potential solution for this the future development of a system for measuring environmental illumination and automatically setting up background luminance in accordance with the measured value. Likewise, it should be considered that different levels of brightness were used on the screens of Optec 6500 and the iPad. However, the contrast sensitivity results obtained were equivalent, and this can be explained because doubling the luminance level improves one letter on a test of five letters per row in the range of 40 cd/m^2^ to 600 cd/m^2^. Although the contrast values on screens of different brightness are similar, the contrast threshold values change with brightness are not similar depending on the spatial frequency [32]; therefore, it is risky to state that the results are completely equivalent. In another study, Kollbaum et al. [33] found results with an iPad-based letter contrast sensitivity test that agreed with those obtained with the Freiburg Acuity and Contrast Test, but it was higher than those measured with the Pelli–Robson Test. These results indicate that the app evaluated was an efficient alternative for clinical use. This parameter was evaluated in 40 subjects (20 with low vision and 20 healthy) in a monocular mode. Likewise, Habtamu et al. [34] developed a new tumbling-E smartphone-based contrast sensitivity test (Peek Contrast Sensitivity, PeekCS) and was compared with a tumbling-E Pelli–Robson contrast sensitivity test. These authors found highly comparable results with both tests.

de Fez et al. [2] used the iPad application “Optopad,” designed to detect color vision deficiencies in two different clinical studies in order to evaluate diagnostic precision. First, a comparison with the Ishihara test was performed in 341 patients (children). A second comparative study with the Farnsworth–Munsell test (FM 100 H) was conducted in 66 patients (university adult population). Previous characterization of the digital device used was made, and the colorimetric adjustments required in the test were performed using the MATLAB software (R2008a) [3,6]. No statistically significant differences were found between “Optopad” and Isihara in detecting protan-deutan defects. When FM 100 H and “Optopad” were compared, a clinically reasonable level of predictability of “Optopad” data from the FM results was observed. Multiple regression analysis provided a regression coefficient (R^2^) of 0.86, with 80% of cases with residuals less than 25 units [2]. This is the second study of validation of a visual function test that provides a complete comparison with conventional tests after a careful calibration of the screens used for the reproduction of stimuli.

Another aspect of the visual function that can be measured with digital applications is the reading speed. Kingsnorth et al. [16] compared reading speed measured by a mobile application and the Radner reading test’s printed version in 21 patients with their usual correction. These authors found that the measurement of reading speed using both methods was not interchangeable. However, the authors concluded that the use of mobile devices was reliable and fast to perform. Notwithstanding, this potential advantage is not altogether valid if the data obtained with this application are not accurate enough.

Finally, a new app has been recently developed for the measurement of the defocus curve with an iPad, which has become an essential clinical test for an adequate evaluation of visual performance with any type of presbyopic correction, including cataract surgery with the implantation of multifocal intraocular lenses [30]. These authors confirmed that the measurement of the visual acuity defocus curve (VADC) showed good agreement with the ETDRS test and good repeatability (two consecutive measurements) despite the short testing time, but it should be mentioned that this interchangeability analysis was performed for visual acuity measures for the level of defocus of 0 D but not for the rest. However, the repeatability of measurements of contrast sensitivity defocus curve (CSDC) was around three times poorer than that obtained along the VADC. The authors concluded that CSDC had to be optimized in the future in order to obtain more repeatable results. In addition to these other articles have been published in recent years, showing the results of the validity of some apps as screening tools used in the evaluation of the visual acuity and visual field in different pathological conditions [35,36,37], but a previous validation in healthy population is crucial.

As mentioned in the introduction part of this study, privacy is a fundamental aspect to consider in the development and operation of a ‘health application,’ since highly sensitive information from patients is used, which must be correctly treated following the current regulation in place. In this manner, the study carried out by Turpin et al. [38] indicated in 2014 that they were subject to American regulations (HIPAA 1996) and that their data will only be uploaded to the cloud if the application (PsyPad) has an internet connection, which shows consistency with the work developed by Benjumea et al. [12] in 2020.

Population selection bias is a critical factor for extrapolating the results of these investigations to the general population, including aspects such as setting (26.3% hospital [1,20,22,28,29], university 21.1% [2,14,18,25]), and ethnicity (26.3%) [15,23,24,26,27] and age (15.8%) [17,20,29]. Likewise, there is a need in some studies to provide separate and adequate description of materials (10.5%) [16,21]. Most of the studies did not report the electronic device model used (commercial model and version of the tablet or iPad). Only a certain level of characterization of the screen used for the generation of the stimuli was performed in two of the studies. However, despite these limitations and the minimal evidence of characterization of digital applications evaluating the visual function, they are widely used in clinical practice and investigations, with the potential of extracting, in some cases, inaccurate conclusions and decisions.

## 5. Conclusions

There are many apps that have been used and validated clinically for measuring different aspects of visual function, such as visual acuity, contrast sensitivity, or color vision. However, limited information is provided in the peer-reviewed literature about the characterization of screens used for displaying stimuli. Therefore, it is not only crucial to use an app validated clinically but also to provide information about the characterization of the devices used for displaying stimuli. One app can be very well designed and validated but used in an electronic device that cannot reproduce the stimuli reliably. For this reason, the technical requirements in terms of screen characteristics must be also provided, and information on how data privacy was obtained must be preserved according to national and international regulations. Thus, an app that is valid for clinical purposes should be validated, and the technical requirements for stimuli reproduction should be well established, allowing the differentiation of well-developed apps from the numerous applications that evaluate visual function and that are available in digital stores. As we are healthcare personnel dealing with patients, the use of previously validated tools (applications) should be mandatory in order to ensure that the results obtained are correct or comparable with conventional methods.

## Figures and Tables

**Table 1:** Summary of the most relevant information of the articles included in the current review.

*Authors*	*Sample*	*Device*	*Apps or Applications*	*Measures*	*Main Results and Conclusions*
Black et al. [14] 2013	*n* = 85 participants	iPad	Visual Acuity XL app	VA (Bailey Lovie and HOTV chart)	➢ With external glare source reflecting over the iPad screen, the iPad (EDTRS chart) provided VA measurements that were significantly worse at an average of 0.18 logMAR
Zhang et al. [15] 2013	*n* = 120 participants	iPad 2	Eye Chart Pro app	VA (E Snellen optotype)	➢ VA median measurements taken with iPad (Eye Chart Pro): 0.40 logMAR
Kingsnorth et al. [16] 2014	*n* = 21 participants	iPad 3	2 custom-made mobile app reading speed charts	Reading speed (Radner reading chart)	➢ ORS custom-made charts: 194 ± 29 wpm and 195 ± 25 wpm; ORS Radner chart: 166 ± 20 wpm; *p* < 0.001. Repeatability: app charts: 0.30 ± 22.5 wpm
Norgett et al. [17] 2014	*n* = 89 participants	iPad 2	Custom-designed visual acuity test	VA (Sloan and ETDRS optotypes)	➢ No statistically significant differences between digital and conventional measurements ➢ VA mean unflanked: 0.0 logMAR
Manzanaro et al. [18] 2015	*n* = 46 participants	iPad	2020 Duo FLEX Visual Acuity Chart	VA (ETDRS optotype)	➢ Significant differences between far VA measured with iPad (2020 Duo FLEX Visual Acuity Chart) and measured with traditional tests ➢ 4 m VA mean; iPad app: 0.093, ETDRS: 0.165; *p* < 0.001; 2 m VA mean; iPad app: −0.089, ETDRS: −0.049; *p* = 0.016
Perera et al. [19] 2015	*n* = 88 participants	iPhone 4	“Snellen” DrBloggs Ltd. app	VA (6 S VA chart)	➢ VA mean difference: 0.02 logMAR (95 % limit of agreement)
Tofigh et al. [20] 2015	*n* = 100 participants	iPhone 5	EyeHand Book app	VA (app vs. Rosenbaum near optotype)	➢ Results could be overestimated as optotypes did not present random-memory effects ➢ VA mean: EyeHand Book app: 0.1398 logMAR SD: 0.132; Rosenbaun optotype: 0.234 logMAR; SD: 0.186
Kingsnorth et al. [21] 2016	*n* = 20 participants	iPad	Aston near app and Aston distance app	CS (CSV-100 and Pelli–Robson tests)	➢ Great repeatability of results obtained with the Aston near and Aston distance apps compared to the CSV-100 test but less than the Pelli–Robson test
Pathipati et al. [22] 2016	*n* = 64 participants	iPhone	Paxos Checkup app	VA (Snellen optotype vs. app)	➢ iPhone measurement was more accurate than non-ophthalmologist healthcare personnel measurement of VA ➢ VA logMAR average: Snellen: 0.211 ± 0.35; *p* = 0.00003 vs. Paxos Checkup: 0.06 ± 0.40; *p* = 0.264)
Phung et al. [23] 2016	*n* = 30 participants	iPad	SightBook mobile app	VA near and far (ETDRS optotype vs. app)	➢ VA measured with both methods differed significantly: they could not be used interchangeably ➢ SightBook VA mean difference: 5.4 letters (RE) and 6.1 letters (LE); Snellen VA mean difference: 7.7 (RE) and 7.9 (LE)
Rhiu et al. [24] 2016	*n* = 43 participants	iPad	iPad-based app: Snellen chart, Tumbling Echart, Landolt C chart, and Arabic figures chart	VA (Snellen, Landolt C optotypes)	➢ Significant correlation between both methods ➢ New method not influenced by the memory effect ➢ Mean logMAR differences: Snellen E: −0.004, Tumbing E: −0.03 and Landolt C: 0.04)
Rodriguez-Vallejo et al. [25] 2016	*n* = 45 participants	iPad	Self-developed app for IOS	VA and CS (ETDRS optotypes)	➢ App comparable with results of OPTHEC6000 ➢ Very practical in clinical use for screening purposes ➢ VA mean differences: 0.06 logMAR (*p* < 0.001) and CS mean differences: 0.05 log units (*p* > 0.05)
Rhiu et al. [26] 2017	*n* = 65 participants	iPad	Korean version reading speed chart	Reading speed	➢ App easy to use, providing results reliable ➢ First app with Korean optotypes to evaluate reading speed ➢ Mean reading speed: 202.3 ± 84.4 wpm and mean reading and speaking speed 129.7 ± 25.9 wpm; *p* < 0.001
de Fez et al. [2] 2018	*n* = 407 participants	iPad	Optopad	Color vision	➢ Comparable diagnostic ability of color vision anomalies compared to Farnsworth–Munsell (FM 100 H) and Ishihara plates.
Bodduluri et al. [27] 2018	*n* = 100 participants	iPad	Three self-developed games	Chromatic contrast sensitivity	➢ Games 1 and 2 and the Cambridge Colour Test (CCT): similar absolute thresholds and tolerance intervals ➢ Game 3: significantly lower values than games 1, 2, and the CCT, due to visual task differences
Azis et al. [28] 2019	*n* = 195 participants	iPad	AAPOS Vision screening app	VA (ETDRS and Sloan optotypes vs. app)	➢ Good correlation between app and conventional optotypes
Brucker et al. [29] 2019	*n* = 120 participants	iPad	Odysight app	VA and CS (app vs. ETDRS test)	➢ Optimal VA measurements ➢ CS results were not as reliable
Fernández et al. [30] 2019	*n* = 127 participants	iPad	Defocus curve app (version 1.0.8)	VA (E Snellen optotype) and CS (CSF test)	➢ Digital measurements quick to do in the clinical setting ➢ Not interchangeable with traditional ones ➢ VA logMAR mean: app: −0.04 ± 0.09 and ETDRS: −0.05 ± 0.08; *p* = 0.51. CS log units mean: app: 0.83 ± 0.23 and CSF: 1.74 ± 0.019; *p* < 0.001
Hogarti et al. [6] 2020	-	iPhone	45 apps (Google play store) and 23 apps (Apple store)	Visual function (mainly VA)	➢ Australian bibliographic review concluding that there is a need to carry out app validations in order to corroborate their effectiveness

Abbreviations: VA, visual acuity; CS, contrast sensitivity; ETDRS, Early Treatment Diabetic Retinopathy Study; FM, Fansworth–Munsell.

## Data Availability

Data are available upon request from the authors.
